# Sacituzumab govitecan as a therapeutic breakthrough in the treatment of triple-negative breast cancer: a systematic review of clinical trials

**DOI:** 10.3389/fimmu.2025.1679648

**Published:** 2026-01-12

**Authors:** Julia Piekarz, Natalia Picheta, Jakub Pobideł, Katarzyna Szklener, Magdalena Skórzewska

**Affiliations:** 1 Student Academic Group, Department of Clinical Oncology and Chemotherapy, Medical University, Lublin, Poland; 2 Department of Clinical Oncology and Chemotherapy, Medical University, Lublin, Poland

**Keywords:** antibody–drug conjugate, metastatic triple-negative breast cancer, sacituzumab govitecan, triple-negative breast cancer, trop-2

## Abstract

**Background:**

Triple-negative breast cancer (TNBC) is one of the most aggressive types of breast cancer (BC), most commonly diagnosed in young premenopausal women. It is characterized by the absence of estrogen receptor (ER), progesterone receptor (PR) and human epidermal growth factor receptor 2 (HER2). For this reason, therapeutic options using hormone therapy or targeted anti-HER2 treatment are significantly limited. TNBC is associated with a poor prognosis, which requires the search for new treatment strategies. A promising option is the use of an antibody-drug conjugate (ADC) directed against Trophoblast cell-surface antigen 2 (Trop-2), namely Sacituzumab govitecan (SG). Recent findings indicate that, beyond its direct cytotoxic effect, SG may also induce immunogenic cell death, remodel the tumor immune microenvironment, and enhance antitumor immune responses—features that make it a molecule of particular relevance in immuno-oncology.

**Materials and methods:**

A systematic review was conducted based on databases from PubMed, Web of Science and the ClinicalTrials.gov registry using the keywords: ‘Sacituzumab govitecan’, ‘Triple-negative breast cancer’, ‘Antibody–drug conjugate’, ‘metastatic triple-negative breast cancer’, ‘Trop-2’. The inclusion criteria covered studies from 2017 to 2025. Ultimately, after a thorough analysis, 4 randomized controlled trials (RCTs) and 4 single-arm studies were included in the review.

**Results:**

Analysis of clinical trial results evaluating the safety and efficacy of SG in TNBC therapy showed promising therapeutic potential for the drug. Significant improvements were observed in key clinical parameters, such as overall response rate (ORR) and progression-free survival (PFS). In addition, the safety profile was acceptable and consistent with previous reports, with the most commonly reported adverse events being neutropenia, diarrhea and alopecia, which were well controlled in most cases.

**Conclusion:**

Due to its aggressive course and poor prognosis, TNBC remains a therapeutic challenge. SG is a promising therapeutic option, but further studies are needed, especially randomized controlled trials and studies on combinations with immunotherapy, to improve treatment outcomes and quality of life for patients. Importantly, SG also exhibits immunomodulatory potential through the induction of immunogenic cell death and enhancement of immune effector activity, positioning it as a bridge between cytotoxic and immune-based therapeutic strategies in TNBC.

## 1 Introduction

Breast cancer (BC) remains one of the leading causes of cancer-related mortality among women worldwide. Among its subtypes, triple-negative breast cancer (TNBC) represents a particularly aggressive form, accounting for approximately 15–20% of all breast cancer cases (1). TNBC is defined by the absence of estrogen receptor (ER), progesterone receptor (PR), and human epidermal growth factor receptor 2 (HER2) expression, which significantly limits treatment options due to the lack of actionable molecular targets (2, 3).

From an immunological perspective, TNBC is unique among breast cancer subtypes due to its relatively high immunogenicity. TNBC tumors are often infiltrated by tumor-infiltrating lymphocytes (TILs), exhibit increased expression of programmed death-ligand 1 (PD-L1), and tend to have a relatively high tumor mutational burden (TMB), making them potentially responsive to immune-based therapies (4). Additionally, germline mutations in BRCA1/2 and defects in DNA damage repair mechanisms contribute to the generation of neoantigens (5). However, the tumor immune microenvironment (TME) in TNBC is highly heterogeneous and frequently dominated by immunosuppressive populations such as regulatory T cells (Tregs) and myeloid-derived suppressor cells (MDSCs), which limit effective immune activation. These dual features—high immunogenic potential and strong immune suppression—make TNBC an attractive yet complex target for immunotherapy (4, 6).

Recent immuno-oncological insights have underscored that TNBC, although aggressive, can serve as a model for exploring the interplay between cytotoxic and immune-modulating therapies. The limited efficacy of immune checkpoint inhibitors alone highlights the need for agents capable of inducing both direct tumor cell death and secondary immune activation (6).

In this context, antibody–drug conjugates (ADCs) have emerged as one of the most promising classes of anticancer agents. ADCs combine the target specificity of monoclonal antibodies with the potent cytotoxicity of chemotherapeutic payloads, delivered directly to tumor cells via synthetic linkers. Their mechanism of action resembles a “Trojan horse” strategy: after binding to a tumor-associated antigen on the cell surface, the ADC is internalized and releases its cytotoxic payload within the cancer cell (7). Beyond their direct cytotoxic effects, several ADCs have been shown to induce immunogenic cell death (ICD)—a form of tumor cell apoptosis characterized by the release of danger-associated molecular patterns (DAMPs), including calreticulin exposure, HMGB1 release, and ATP secretion. These signals promote dendritic cell maturation and T-cell priming, thus linking ADC-mediated cytotoxicity with adaptive immune activation (8).

Given these immunomodulatory properties, ADCs may represent a new therapeutic bridge between traditional chemotherapy and immunotherapy. Sacituzumab govitecan (SG) exemplifies this dual potential. SG is an ADC composed of a humanized IgG1 monoclonal antibody targeting trophoblast cell-surface antigen 2 (Trop-2)—a 35-kDa transmembrane glycoprotein overexpressed in several epithelial malignancies, including TNBC—linked to the active metabolite of irinotecan, SN-38, via a hydrolyzable CL2A linker. Beyond its established cytotoxic mechanism, SG also modulates immune processes within the TME (9).

Recent preclinical studies suggest that SN-38, the payload of SG, not only induces DNA damage but also contributes to immunogenic modulation by upregulating MHC class I expression on tumor cells and repolarizing macrophages toward a proinflammatory M1 phenotype. These mechanisms may enhance antigen presentation and facilitate T-cell infiltration within the tumor microenvironment (10).

The clinical efficacy of SG has been confirmed in pivotal studies, such as ASCENT, and ongoing research explores its use in combination with checkpoint inhibitors—further emphasizing its immunotherapeutic relevance (11).

Taken together, SG represents a bridge between classical cytotoxic therapy and modern immunotherapy. Its ability to induce immunogenic cell death and reshape the tumor immune microenvironment provides a strong rationale for integrating SG into future immuno-oncology strategies for TNBC (12).

Therefore, the aim of this review is to provide an up-to-date evaluation of both the clinical efficacy and the emerging immunological mechanisms of sacituzumab govitecan in TNBC, with particular consideration of results from recent clinical and translational studies.

## 2 Materials and methods

### 2.1 Study designs

This systematic review was conducted in accordance with the Preferred Reporting Items for Systematic Reviews and Meta-Analyses (PRISMA) 2020 guidelines. The aim of the review was to comprehensively assess the efficacy and safety of SG in the treatment of TNBC, both as monotherapy and in combination with immunotherapy. The analysis included different types of studies, primarily randomized controlled trials (RCTs) and single-arm studies, to provide a broad perspective on available evidence.

### 2.2 Eligibility criteria and PICO framework

The research focused on evaluating the efficacy and safety of the therapy. The PICO model was used to structure and focus the literature review.

- Population: The target population consists of adult patients suffering from TNBC, regardless of gender.
- Intervention: interventions include the use of SG monotherapy or a combination of SG and immunotherapy.
- Comparison: A comparative analysis of SG therapy with other therapeutic regimens was performed where data were available. For single-arm studies, results were analyzed without comparison with a control group.
- Outcome: The results analyzed in the review focused on evaluating the efficacy and safety of therapy with SG, either as monotherapy or in combination with immunotherapy, in patients with TNBC. Efficacy was mainly assessed by objective response rate (ORR), disease progression-free time (PFS), overall survival (OS), duration of response (DoR) and other clinical parameters when available. Safety of therapy was analyzed by assessing the incidence of Treatment-Related Adverse Events (TRAEs), including serious adverse events (≥ grade 3).

Studies published in English between 2017 and 2025 were included in the analysis. RCTs and single-arm studies were included if they contained data on the use of SG in the patient population in question. Exclusion criteria included review publications, reports of preclinical studies (both in animal and cellular models), abstracts without complete data (e.g., conference abstracts or posters that did not provide full results), and papers that did not include information on the efficacy or safety of Sacituzumab govitecan treatment.

### 2.3 Search search and selection process

The search terms in the databases: PubMed, Web of Science and ClinicalTrials.gov registry covered the years 2017–2025 to provide the most up-to-date data. Keywords used: “Sacituzumab govitecan”; “Triple-negative breast cancer”; “Antibody–drug conjugate”; “Metastatic Triple-negative breast cancer”; “Trop-2”. To ensure the completeness of the review, a manual search of the results and an evaluation of the bibliographies of the selected articles was also conducted. Two researchers (J.P. and K.S.) independently searched the databases and critically appraised the selected articles. Any discrepancies were resolved by verification by two other authors (N.P. and S.M.).

**Figure 1:**
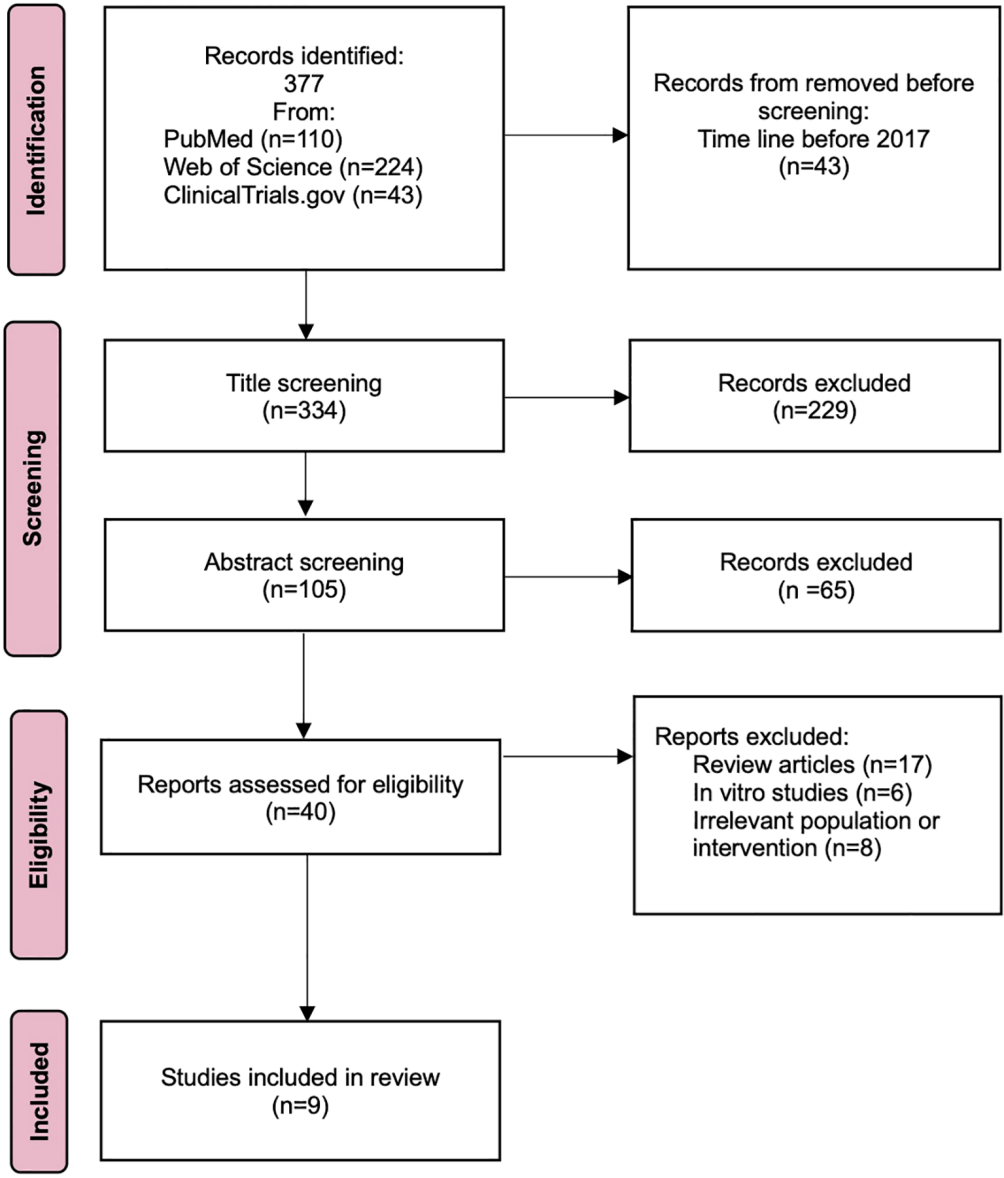
Preferred Reporting Items for Systematic Reviews and Meta-analyses (PRISMA) flow diagram of study identification, inclusion, and exclusion.

A systematic database search identified 377 records, of which, 43 records were excluded for not meeting the temporal inclusion criteria, and 294 records were omitted due to irrelevance of outcomes or results. An additional 31 records were excluded as they comprised review articles, *in vitro* studies and irrelevant population or intervention. Ultimately, 9 articles met the inclusion criteria and were incorporated into the primary analysis. Following a quality assessment, all 9 studies were deemed to be of high methodological quality. The comprehensive data selection and identification process is illustrated in **Figure 1**.

Although the keyword “ADC” may appear broad, it was intentionally included to ensure that all Trop-2–targeting ADCs, including SG, were captured. This approach minimized the risk of omitting relevant studies and reflects the limited number of available clinical trials rather than overly restrictive criteria.

### 2.4 Data extraction

The review included five RCTs and four single-arm studies involving a total of 2059 participants diagnosed with TNBC and treated with SG. Data extraction was performed independently by two reviewers using a standardized data extraction form. Extracted data included study characteristics (author, year, study design), patient demographics (total number of patients, age, gender), intervention details (SG dosage, regimen, monotherapy vs. combination therapy), and outcome measures related to efficacy (ORR, PFS, OS, DoR, Complete Response (CR), Partial Response (PR), Stable Disease (SD), Progressive Disease (PD), Clinical Benefit (CD) and safety (incidence and severity of TRAEs). Any discrepancies were resolved through discussion or consultation with a third reviewer.

### 2.5 Assessment of risk of bias in the included studies

The included studies comprised RCTs and single-arm studies.

For the RCTs, risk of bias was assessed using the updated Cochrane Risk of Bias tool for randomized trials (RoB 2.0) (Higgins, 2019). This tool evaluates five domains: (1) bias arising from the randomization process, (2) bias due to deviations from intended interventions, (3) bias related to missing outcome data, (4) bias in outcome measurement, and (5) bias in the selection of reported results. Two independent reviewers applied RoB 2.0 to all included RCTs, recording supporting evidence and providing judgments of low risk, high risk, or some concerns for each domain. Discrepancies were resolved by discussion or adjudication by a third reviewer.

For the single-arm studies, risk of bias was assessed using the NIH Quality Assessment Tool for Before-After (Pre-Post) Studies with No Control Group. This tool examines key aspects such as selection bias, confounding variables, outcome assessment, and reporting bias to evaluate study quality.

This tailored approach to risk of bias assessment, specific to each study design, ensured a comprehensive evaluation of study quality and enhanced the overall reliability of the review findings.

## 3 Triple-negative breast cancer

TNBC is a subtype of BC characterized by the absence of ER, PR and HER2 receptor expression. In addition, it is characterized by a high histological grade and a Ki-67 proliferation index exceeding 80%, as well as molecular and biological heterogeneity (13). The combination of these tumor characteristics makes it the most aggressive subtype of BC, limiting effective treatment options and resulting in poor prognosis, increased risk of recurrence and distant metastasis, and low survival rates (14).

TNBC accounts for approximately 15-20% of all BC cases (15). It is diagnosed much more frequently in premenopausal women, especially in patients under 50 years of age. This phenomenon highlights the need to expand knowledge about TNBC as a distinct clinical and biological entity (16). In addition, it is diagnosed much more frequently in patients with BRCA1 mutation and African American women (17). The 5-year OS for metastatic TNBC is approximately 11%, and the median OS is approximately 11–13 months (18). In addition, a high level of cell invasiveness and visceral metastases to organs, primarily the brain, lungs and liver, are observed. Metastatic TNBC (mTNBC) has a significantly shorter survival period after distant metastases occur (19).

At the molecular level, TNBC is divided into subtypes, including basal-like 1/2, immunomodulatory, mesenchymal, mesenchymal stem-like, and luminal androgen receptor (LAR), each of which exhibits a different response to therapy and potential molecular targets (20).

The diagnosis of TNBC is based on immunohistochemical (IHC) examination of a tumor biopsy, which confirms the absence of ER, PR (below 1%) and HER2 expression (0 or 1+, or no amplification in FISH with HER2 2+) (21). In addition, the Ki-67 index is assessed, BRCA1/2 mutations are evaluated, and in some cases, tumor genetic profiling is also performed, especially in the context of qualification for systemic treatment (22).

TNBC also exhibits relatively high immunogenicity (including higher lymphocyte infiltration density – TILs – and a higher likelihood of PD-L1 expression), which makes this subtype particularly interesting from the perspective of the development of immunotherapies and ADCs (23).

Currently, chemotherapy remains the first-line treatment for TNBC and is the standard therapeutic strategy for both locally advanced and metastatic disease. In mTNBC, the response to chemotherapy is usually limited, and treatment outcomes do not meet expectations for survival prolongation (24). Monochemotherapy in mTNBC patients is associated with an ORR of 25–35%, moderate PFS (median PFS below 6 months) and a median OS of less than 12 months (25). The characteristic lack of expression of hormone receptors (ER, PR) and HER2 receptors excludes the possibility of hormone therapy and HER2-targeted therapy, which significantly limits the available targeted treatment options. In addition, the potential use of PARP inhibitors and checkpoint inhibitor-based immunotherapy is increasingly being suggested (26).

In recent years, the dynamic development of immunotherapy and ACD-based therapies has opened up new therapeutic perspectives. Nevertheless, there are still many challenges associated with improving the effectiveness of treatment and the quality of life of patients affected by this aggressive subtype of cancer (27).

## 4 Sacituzumab govitecan

ADC is a modern form of cancer therapy that combines the high specificity of monoclonal antibodies with the potency of highly cytotoxic drugs. Structurally, ADCs consist of three key elements: an antibody directed against a specific tumor antigen, a payload, i.e., a cytotoxic substance, and a linker that enables stable binding of the drug to the antibody and its selective release in the target cell. This allows ADCs to precisely deliver the drug directly to cancer cells, limiting damage to healthy tissue and minimizing systemic toxicity (28).

ADCs are currently used in the treatment of various cancers, both solid and hematological (29). Their action is based on three main mechanisms: the effect of targeted drug delivery – after binding to the antigen on the surface of the cancer cell, the ADC is internalized and the payload is released inside the cell, the so-called bystander effect – some payloads, such as SN-38, can penetrate neighboring cells, destroying even those that do not express the antigen in question, and potential immunomodulation – there is evidence that some ADCs can affect the tumor microenvironment, increasing its immunogenicity and enhancing the antitumor response (30).

The most commonly used payloads include microtubule inhibitors such as monomethyl auristatin E (MMAE) or monomethyl auristatin F (MMAF), alkylating agents such as calicheamicin, and topoisomerase I inhibitors such as the active metabolite of irinotecan (SN-38) or exatecan, a synthetic derivative of camptothecin. The choice of payload depends on the type of cancer, its sensitivity to a given mechanism of action, and pharmacokinetic properties (31).

One of the most promising ADCs used clinically is SG. It consists of an IgG1 monoclonal antibody hRS7 directed against Trop 2, which is widely expressed in tumors, including TNBC, bladder, lung, and cervical cancer. SN-38 is attached to the antibody using a cleavable linker (CL2A). It is distinguished by a relatively high DAR of approximately 7.6, which increases the amount of drug delivered to the tumor cell (32).

**Figure 2:**
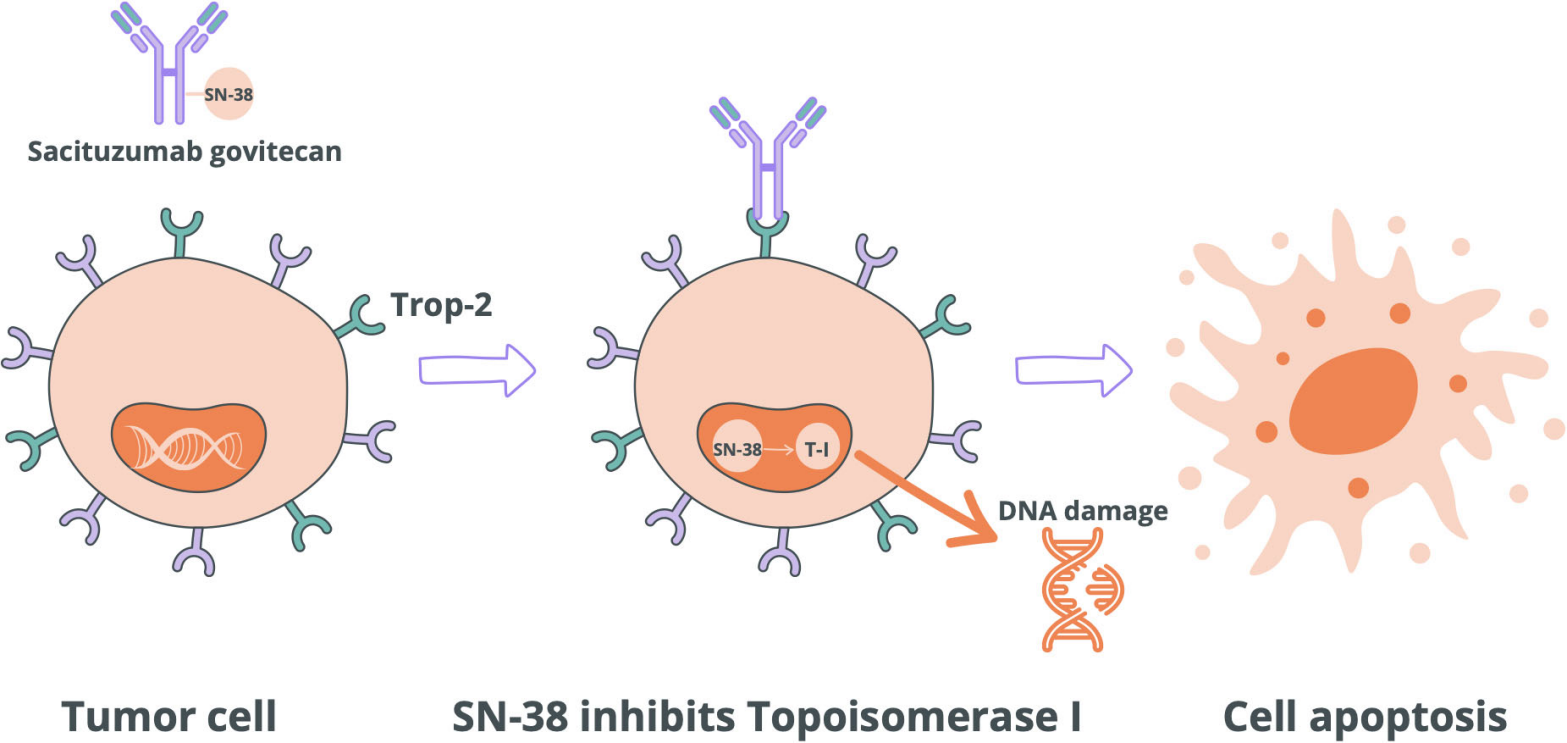
Mechanism of action of SG. Topoisomerase I (T-I).

The mechanism of action of SG is based on the specific binding of the antibody to Trop-2, internalization of the complex, and release of SN-38 inside the cell. In the context of TNBC, this mechanism is particularly relevant because TNBC cells often exhibit high expression of Trop-2, making them sensitive targets for SG. Clinically, sacituzumab govitecan is indicated for patients with metastatic or locally advanced, unresectable TNBC who have experienced disease progression after at least two prior systemic chemotherapy regimens. Therefore, SG is primarily used in advanced or metastatic stages of TNBC, when resistance to standard chemotherapy develops and treatment options become limited (32). Importantly, due to its mechanism and the bystander effect, SG may retain therapeutic activity even in patients with visceral or distant metastases, including lesions in the lungs or central nervous system. Its targeted action enables effective cytotoxic delivery even in heavily pretreated patients, where conventional chemotherapy shows limited benefit. After activation in an acidic environment, SN-38 binds to topoisomerase I, leading to DNA breaks and cell death. In addition, due to the diffusion of SN-38, a bystander effect is observed – a cytotoxic effect on neighboring tumor cells, even if they do not express Trop-2 (33). The mechanism of action of SG is shown in **Figure 2**.

SG has been approved by the Food and Drug Administration (FDA) for the treatment of TNBC after prior chemotherapy (ASCENT trial) and for hormone-dependent HER2-negative breast cancer. The ASCENT trial demonstrated a significant prolongation of PFS 5.6 vs. 1.7 months and OS 12.1 vs. 6.7 months compared to chemotherapy (34). Clinical trials are currently underway to investigate the use of SG in the treatment of other cancers, including small cell lung cancer (SCLC), non-small cell lung cancer (NSCLC), cervical cancer, and bladder cancer. Metastatic spread, particularly to the brain and lungs, remains a major therapeutic challenge in TNBC, as the efficacy of SG may be influenced by biological barriers such as the blood–brain barrier and by organ-specific microenvironments. Brain metastases pose a special problem due to the limited permeability of the blood–brain barrier (BBB); however, some clinical observations suggest that partial penetration of SN-38 and the bystander effect may provide limited activity in intracranial lesions. Lung metastases, on the other hand, tend to be more accessible to systemic ADC therapy, and responses have been documented in pulmonary lesions. Further research is needed to determine the extent to which SG crosses the BBB and retains activity in the central nervous system (28).

SG was first approved by the FDA on April 22, 2020, under an accelerated approval pathway for the treatment of mTNBC after ≥2 lines of therapy. On April 7, 2021, this approval was confirmed by full registration based on the results of the ASCENT Phase III trial. On April 13, 2021, the drug received accelerated approval for urothelial cancer after prior chemotherapy and immunotherapy. The most recent indication expansion took place on February 3, 2023, when the FDA approved it for the treatment of hormone-dependent, HER2-negative breast cancer (HR+/HER2-) after ≥2 lines of systemic therapy (35). Based on available studies, the recommended dose of SG is 10 mg/kg body weight, administered intravenously on days 1, 8, and 15 of each 21-day treatment cycle (36).

In terms of safety, SG most commonly causes hematologic toxicities, including neutropenia, anemia, leukopenia, and thrombocytopenia, which can increase the risk of infections and may require supportive therapy such as G-CSF. Gastrointestinal adverse events, such as diarrhea, nausea, vomiting, and abdominal discomfort, are frequently observed and may require antidiarrheal agents, antiemetics, and careful monitoring of hydration and electrolytes. Fatigue and asthenia are commonly reported and can affect patient quality of life. Less common adverse events include rash, dry skin, elevated liver enzymes, and neurological symptoms, particularly in patients with CNS metastases (33). In patients with UDP-glucuronyltransferase 1A1 (UGT1A1*28) gene polymorphism or hepatic impairment due to metastases may have a higher risk of toxicity, emphasizing the importance of genotyping and close clinical monitoring. Overall, with appropriate supportive care and dose adjustments, SG maintains a manageable safety profile even in heavily pretreated metastatic TNBC patients (37).

The future of SG and other ADCs lies in the search for new therapeutic indications, increasing efficacy through combinations with PARP inhibitors (talazoparib) or other ADCs, and research into the role of ADCs in immunomodulation. The development of new linking technologies, better selection of biomarkers, and understanding of resistance mechanisms may allow for even more precise and effective cancer therapies (30).

## 5 Immunological aspects of sactituzumab govitecan in triple-negative breast cancer

Beyond their direct cytotoxic potential, ADCs such as SG have emerged as potent immunomodulatory agents, capable of reshaping the tumor immune microenvironment (TME) and potentiating antitumor immune responses. One of the pivotal mechanisms through which ADCs may exert such effects is the induction of immunogenic cell death (ICD)—a form of regulated cell death that facilitates dendritic cell maturation and the priming of cytotoxic T lymphocytes (38). Preclinical studies have demonstrated that SN-38, the active payload of SG, can induce key hallmarks of ICD, including surface exposure of calreticulin (CRT), extracellular release of high-mobility group box 1 (HMGB1), and ATP secretion (39). These molecular signals serve as “danger-associated molecular patterns” (DAMPs), promoting efficient antigen uptake and presentation (40). Comparative analyses suggest that topo-isomerase I inhibitors, particularly when delivered via ADCs, may enhance ICD more robustly than their unconjugated counterparts, likely due to improved tumor selectivity and intracellular retention. These features position SG as not only a cytotoxic agent, but also a functional enhancer of innate immune recognition (9).

The immunomodulatory actions of SG extend further into remodeling the TME. In murine TNBC models, administration of SG was associated with significant phenotypic changes in tumor-associated macrophages (TAMs), skewing their profile from an M2-like, immunosuppressive phenotype towards an M1-like, pro-inflammatory and antigen-presenting state (41). This switch is critical for restoring local immune surveillance and facilitating T-cell recruitment. Furthermore, SG treatment has been shown to reduce the abundance of immunosuppressive regulatory T cells (Tregs) and myeloid-derived suppressor cells (MDSCs), both of which play central roles in shielding tumors from immune-mediated clearance (42). Parallel to the reduction of these suppressive populations, SG enhances the infiltration and activation of CD8^+^ cytotoxic T lymphocytes (CTLs). These T cells exhibit upregulation of key effector molecules such as granzyme B and interferon-γ, suggesting not only increased presence but also functional activation of the antitumor immune arm. Collectively, these alterations in cellular composition and activation status of the TME indicate that SG acts to reverse immune exclusion and reinvigorate adaptive immune responses within the tumor bed (9).

On the mechanistic level, SN-38 appears to modulate antigen presentation pathways directly (43). *In vitro* and *in vivo* studies have shown that SG increases the expression of major histocompatibility complex class I (MHC-I) molecules on tumor cells, thereby enhancing the visibility of malignant cells to CD8^+^ T cells. This upregulation is accompanied by modulation of the cytokine milieu, favoring a pro-inflammatory and T cell-permissive environment (44, 45). In some experimental systems, these changes have been linked to the facilitation of epitope spreading—a process wherein immune responses are broadened beyond the initial tumor antigens. Although specific data regarding neoantigen expression post-SG therapy remain sparse, the intrinsic genomic instability of TNBC and the DNA-damaging effects of SN-38 suggest a plausible increase in neoepitope availability. Trop-2, the target antigen of SG, is not classically immunogenic; however, its restricted expression in normal tissues and high-level overexpression in malignant epithelial cells make it a rational surrogate for immune recognition in the presence of ADC-induced immunologic stress (46). Trop-2 may also act as a carrier facilitating cross-presentation of tumor-associated peptides, although this remains an area of active investigation (47).

Immunotoxicity is an important consideration in evaluating the immunologic footprint of SG. The most frequently observed immune-related adverse event is neutropenia, which can transiently compromise innate immune function and increase susceptibility to infection (48). However, this toxicity is generally manageable with supportive care and does not appear to significantly impair antigen-specific T cell responses or hinder the development of immunologic memory. In fact, emerging data suggest that SG may be safely combined with immune checkpoint inhibitors (ICIs), such as PD-1 or PD-L1 blockade, without exacerbating toxicity profiles—a hypothesis currently under clinical evaluation (49).

In summary, sacituzumab govitecan represents a new generation of ADCs with dual capacity: direct induction of tumor cell death and active reprogramming of the immune microenvironment. Its ability to induce ICD, shift macrophage polarization, reduce immunosuppressive cell subsets, enhance antigen presentation, and potentially facilitate neoantigen-driven immunity provides strong rationale for its integration into immunotherapy-based regimens. As our understanding of ADC immunobiology deepens, SG may serve as a paradigm for combining targeted cytotoxicity with immune potentiation in the treatment of immunologically cold tumors such as TNBC.

## 6 Clinical results

### 6.1 Phase I/II study IMMU-132-01 (NCT01631552)

The open-label, single-arm Phase I/II study included 69 women with mTNBC who had previously undergone a minimum of 5 lines of treatment on average. Patients received SG administered according to the standard treatment protocol. The results proved extremely promising for such a therapy-resistant group of patients. The median DoR was 8.9 (95% CI, 6.1 to 11.3 months), while the median PFS was 6 months (95% CI, 5.0 to 7.3 months) and the median OS was 16.6 months (95% CI, 11.1 to 20.6 months). **Table 1** presents numerous study results indicating the efficacy of SG in the treatment of mTNBC. Although this is only a phase I/II study, it provides a solid basis for further research and the establishment of new treatment standards (50).

**Table 1:** Results of Phase I/II study IMMU-132-01 (NCT01631552) evaluating the efficacy of SG in patients with mTNBC.

Clinical parameter	SG group
SD	45%
PD	25%
CR	3%
PR	28%
ORR	30%
CB	46%

Dose SG: 10 mg/kg on days 1, 8, and 21 of the cycle (50).

### 6.2 Multicenter phase IIB study (NCT03964727)

A multicenter, open-label, single-arm Phase IIB study enrolled 80 patients with advanced TNBC or mTNBC who had previously received at least two lines of treatment. Patients received SG administered according to the standard treatment protocol. The results showed that the treatment was highly effective in a difficult patient population with a poor prognosis and limited response to other therapies. The results are presented in **Table 2**. In addition, Sg proved to be safe, as only 6.3% of patients discontinued treatment due to TEAEs. The most commonly diagnosed hematopoietic disorders were neutropenia (85%), anemia (82.5%), leukopenia (81.3%), vomiting (55%), and nausea (50%). In addition, decreased appetite was diagnosed in 26.3%, fatigue in 22.5%, and alopecia in 33.8% (51).

**Table 2:** Results of Multicenter Phase IIB Study (NCT03964727) evaluating the efficacy of SG in patients with TNBC or mTNBC.

Clinical parameter	SG group
ORR	38.8%
CR	2.5%
PR	36.3%
SD	43.8%
DCR	82.5%
CBR	43.8%
PFS	5.5 months

Dose SG: 10 mg/kg on days 1, 8, and 21 of the cycle (51). Clinical Benefit Rate (CBR).

### 6.3 NeoSTAR phase II study (NCT 04230109)

The NeoSTAR study evaluated neoadjuvant treatment with SG. It is a single-arm phase II study that enrolled 50 patients with localized TNBC in stages I-III (26% in stage I, 52% in stage II, and 22% in stage III). The results were incredibly promising for monotherapy and showed that pathological complete response (pCR) was achieved in 30% of patients. The highest pCR was found in stage I patients, where it was 50%. In stage II, it was 27%, and in stage III, 18% of patients. ORR was 64%. CR or PR was achieved in 27 patients, SD in 12 women, and PD in only 1. Long-term follow-up of 18.9 months showed that the 2-year PFS of all women was 95% (95% CI 88% to 100%). Although this is not an RCT, it is worth including it in this systematic review due to the exceptionally promising results of preoperative monotherapy with SG and to emphasize the need for further development and expansion of clinical trials in this therapeutic area (52).

### 6.4 Multicenter phase IIb study (NCT01631552)

A multicenter, open-label, single-arm study included 107 women and 1 man. Each patient was diagnosed with mTNBC and underwent between 2 and 10 lines of therapy. The results showed that SG monotherapy at the standard protocol dose is a promising therapeutic method. The ORR was 33.3% (95% CI 24.6–43.1), PR was achieved in 3 patients, and PR 33. The clinical benefit rate was 45.4%. The median DoR was 7.7 months (95% CI 4.9–10.8). The median PFS was estimated at 5.5 months (95% CI 4.1–6.3), and the median OS was 13 months (95% CI 11.2–13.7) (53).

### 6.5 ASCENT phase III trial (NCT02574455)

The ASCENT study included 468 patients with mTNBC, but without brain metastases, who had relapsed or were resistant to at least two standard chemotherapy regimens. 235 patients received SG at the standard protocol dose. In contrast, 233 women received standard chemotherapy based on one of four drugs: eribulin (1.4 or 1.24 mg/m2 intravenously on days 1, 8, and 21 of the cycle), vinorelbine (25 mg/m2 intravenously on day 1 weekly), calepcytamine (1000–1250 mg/m2 orally twice daily for 14 days with a 7-day break) or gemcitabine (800–1200 mg/m2 intravenously on days 1, 8, and 21 of the cycle). In the SG group, the median PFS was 5.6 months (95% confidence interval (CI), 4.3 to 6.3), while in the chemotherapy group it was 1.7 months (95% CI, 1.5 to 2.5). The PFS hazard ratio was 0.41 (95% CI, 0.32-0.52, p<0.00). The median OS was 12.1 months (95% CI, 10.7-14.0) in the study group, but 6.7 months (95% CI, 5.8-8.7) in the control group. The hazard ratio (HR) was 0.48 (95% CI, 0.38-0.59). The rest of the results indicating a promising SG efficacy profile in patients with TNBC are presented in **Table 3** (54).

**Table 3:** Results of ASCENT Phase III Trial (NCT02574455) of efficacy of SG in patients with mTNBC (54).

Clinical parameter	Study group	Control group
ORR	35%	5%
CR	4%	1%
PR	31%	4%
SD	34%	27%
PD	23%	38%
CB	45%	9%

### 6.6 ASCENT safety substudy (NCT02574455)

The study evaluated the safety of SG compared to traditional chemotherapy. A total of 340 patients with a primary diagnosis of TNBC participated in the study. SG at the standard protocol dose was administered to 184 patients, while TPC (eribulin, vinorelbine, gemcitabine, or capecitabine) was administered to 156 patients. The safety of ADC in TNBC therapy was analyzed. The analysis showed that TRAEs were diagnosed in over 20% of patients, while grade 3 TRAEs were diagnosed in approximately 5% of patients. The most commonly diagnosed side effects were nausea, diarrhea, neutropenia, fatigue, and alopecia. The detailed results are presented in **Table 4** and indicate the safety of SG use. Perhaps the percentage was lower than that of TPC (55).

**Table 4:** TRAEs in the ASCENT Substudy (NCT02574455) assessing SG safety (55).

TRAE (%)	SG group			TPC group		
	All	Level 3	Level 4	All	Level 3	Level 4
Neutropenia	59%	33%	15%	41%	18%	12%
Anemia	36%	8%	0%	24%	4%	0%
Leukopenia	16%	8%	1%	10%	4%	0%
Neutropenic fever	7%	5%	2%	2%	1%	1%
Fatigue	42%	4%	0%	29%	4%	0%
Decreased appetite	17%	2%	0%	13%	1%	0%
Alopecia	46%	0%	0%	19%	0%	0%
Nausea	55%	2%	1%	26%	0%	0%
Vomiting	29%	1%	1%	10%	0%	0%
Diarrhea	58%	12%	0%	12%	1%	0%

### 6.7 ASCENT quality-of-life substudy (NCT02574455)

The RCT evaluated the quality of life of 419 patients with recurrent or refractory mTNBC after a minimum of two lines of therapy. 236 received SG and 183 received TPC. Quality of life was assessed using the European Organisation for Research and Treatment of Cancer Quality of Life Questionnaire – Core 30 (EORTC QLQ-C30), which is a validated tool for measuring the health status and quality of life of cancer patients. This questionnaire consists of 30 items and allows for the assessment of Global Health Status/Quality of Life, a functioning scale, and a symptom scale. The results presented in **Table 5** showed a significant delay in the deterioration of quality of life in patients treated with SG, especially in the areas of physical functioning, fatigue, and pain. This confirms the longer time to deterioration of well-being in the SG group (56).

**Table 5:** Time to first clinically significant deterioration in quality-of-life parameters in patients with mTNBC in the ASCENT QoL Substudy (NCT02574455) (56).

Domain assessed	SG group	TPC group	Value p
Physical functioning	22.1 weeks	12.1weeks	<0,001
Fatigue	7.7 weeks	6.0 weeks	<0,05
Pain	21.9 weeks	9.9 weeks	<0,001
Role functioning	11.4 weeks	7.1 weeks	<0,001

### 6.8 SACI-IO TNBC trial (NCT04468061)

A phase II randomized study evaluated the potential efficacy of combining ADC drugs with immunotherapy. SG was used in combination with pembrolizumab, which is a PD-1 receptor inhibitor on immune system cells, enhancing the antitumor response. Eighty-two patients were enrolled and divided into two groups: 52 patients received SG + pembrolizumab (SG+PE), while 30 received SG monotherapy. The ORR in the SG group was 17.3%, while in the SG+PE group it was 21.2%, CBR was 46.2% and 50%. The results showed that adding immunotherapy to SG produces better results and may enhance the effect of SG and improve patient prognosis (57).

### 6.9 ASCENT-04/KEYNOTE-D19 phase III (NCT05382286)

The ASCENT-04/KEYNOTE-D19 was a randomized, open-label, phase III trial evaluating SG in combination with pembrolizumab versus pembrolizumab plus chemotherapy in patients with previously untreated, PD-L1–positive, locally advanced or mTNBC. A total of 443 patients were enrolled and randomized in a 1:1 ratio to receive either SG at the standard protocol dose plus pembrolizumab (200 mg every 3 weeks) (n = 221) or pembrolizumab combined with the physician’s choice of chemotherapy—nab-paclitaxel, paclitaxel, or gemcitabine/carboplatin (n = 222). The combination of SG + pembrolizumab demonstrated a statistically significant and clinically meaningful improvement in PFS, with a median PFS of 11.2 months (95% CI 9.3–16.7) compared with 7.8 months (95% CI 7.3–9.3) in the chemotherapy + pembrolizumab arm (HR 0.65; p < 0.001). The ORRreached 62% (95% CI 56–68) in the SG + pembrolizumab arm compared with 40% (95% CI 34–46) in the control arm. The median DOR was 16.5 months (95% CI 12.1–not reached) versus 9.8 months (95% CI 7.4–11.6), respectively.

The safety profile of the combination was consistent with prior experience for each agent. The most common TRAEs included neutropenia, diarrhea, and fatigue. Grade ≥ 3 neutropenia occurred in 52% of patients, diarrhea in 10%, and anemia in 8%. Treatment discontinuation due to adverse events was infrequent (7%). These results confirm the feasibility and clinical benefit of combining ADC-based therapy with immune checkpoint inhibition in the first-line treatment of PD-L1–positive mTNBC (58).

### 6.10 Comparative overview of efficacy and safety

Across clinical studies, SG showed consistent efficacy and manageable safety in patients with TNBC. In metastatic settings, median PFS ranged from 5–6 months, and OS from 12–16 months, with ORR around 30–35%, markedly higher than with standard chemotherapy.

In the neoadjuvant NeoSTAR trial, SG achieved an ORR of 64% and pCR of 30%, while ASCENT-04/KEYNOTE-D19 confirmed enhanced activity when combined with pembrolizumab, suggesting a potential immunologic synergy.

Adverse events were predictable and manageable, with neutropenia and diarrhea as the most common. Severe toxicities and treatment discontinuations were infrequent.

Overall, SG provides a reproducible clinical benefit and favorable safety profile, representing a bridge between targeted cytotoxic therapy and immune modulation in TNBC.

**Table 6** summarizes the key studies included in this review, confirming the clinical benefits of SG in TNBC therapy.

**Table 6:** Summary table of studies evaluating the efficacy and safety of SG in TNBC therapy.

Year	Type of examination	Intervention	Population	Results	Citation
2017	Single-arm trials	SG	69	ORR 30% PFS median 6 months OS median 16.6 months DoR median 10.2 months	(50)
2023	Single-arm trials	SG	80	ORR 38.5% PFS median 5.5 months DCR 82,5%	(51)
2024	Single-arm trials	SG	50	ORR 64% pCR 30% 24 months PFS 95%	(52)
2019	Single-arm trials	SG	108	ORR 33.3% PFS median 5.5 months OS median 13 months	(53)
2021	RCTs	SG	468	PFS median 5.6 months OS median 12.1 months ORR 35%	(54)
2022	RCTs	SG	340	Most TRAEs were mild to moderate; effective monitoring limited toxicity.	(55)
2023	RCTs	SG	419	SG delayed QoL deterioration, especially in physical function, fatigue, and pain.	(56)
2021	RCTs	SG+PE	82	ORR 21.2% CBR 50%	(57)
2025	RCTs	SG+PE	443	PFS median 11.2 months ORR 59.7% DOR median 16.5	(58)

### 6.11 Gaps and future directions

Despite the significant progress achieved with SG, several key gaps remain that must be addressed to optimize its use and fully realize its therapeutic and immunologic potential in TNBC.

First, biomarker identification remains a major unmet need. Although SG targets Trop-2, the correlation between Trop-2 expression levels and treatment outcomes has not been clearly established. The extreme molecular heterogeneity of TNBC—including basal-like, mesenchymal, and immunomodulatory subtypes—suggests that not all patients benefit equally. Future translational studies should focus on defining predictive markers such as Trop-2 intensity, homologous recombination deficiency (HRD) status, tumor-infiltrating lymphocytes (TILs), and immune gene expression profiles to improve patient selection and guide personalized therapy.

Another important aspect is that most available data come from studies involving patients with advanced, heavily pretreated TNBC, while the potential of SG in earlier disease stages is only beginning to be explored. Several ongoing clinical trials are currently evaluating SG in neoadjuvant and adjuvant settings (e.g., NCT04230109, NCT06081244), as well as in first-line combinations with immunotherapy. Among them, NCT04468061 investigates SG combined with pembrolizumab, NCT07040644 explores its combination with toripalimab, and NCT04434040 evaluates SG together with atezolizumab. These studies aim to determine whether earlier integration of SG and its combination with immune-modulating therapies can improve long-term disease control, reduce recurrence risk, and extend survival in patients with TNBC.

In addition, mechanisms of resistance remain poorly understood. Possible contributors include antigen loss, efflux transporter upregulation, or changes in TME that impair payload delivery. Preclinical studies are needed to clarify how repeated SG exposure reshapes immune infiltration, macrophage polarization, and cytokine signaling, and how these effects may be leveraged to overcome resistance or enhance immunotherapy efficacy.

Another area requiring attention is the limited availability of real-world evidence (RWE). Data from routine clinical practice, including elderly and comorbid patients, are necessary to confirm the reproducibility of clinical outcomes outside of controlled trials. Real-world cohorts could provide valuable insights into the management of older and comorbid TNBC patients, who are typically underrepresented in randomized trials. Such studies would also allow assessment of cumulative toxicity, treatment sequencing, and long-term quality-of-life outcomes in broader populations. Such studies will also provide insights into long-term safety, cumulative toxicity, and quality-of-life impacts, which are difficult to assess in short-duration randomized trials.

Looking ahead, the future of SG likely lies in its integration into immuno-oncology frameworks. Given its ability to induce IC and alter the tumor immune microenvironment, SG has the potential to act as an immune sensitizer. Rationally designed combinations with PD-1/PD-L1, CTLA-4, or STING agonists could potentiate durable immune activation and prevent relapse. Advances in ADC engineering, including alternative payloads or dual-target constructs, may further expand the scope of Trop-2–directed therapies across TNBC subtypes and even other solid tumors.

In conclusion, the next phase of SG research should focus on biomarker-driven clinical trials, mechanistic immunology, and real-world validation to refine its therapeutic positioning. A deeper understanding of how SG interacts with the immune system and other targeted treatments will be essential to transform it from a promising option into a cornerstone of personalized, immune-integrated TNBC therapy.

## 7 Discussion

TNBC remains one of the most therapeutically challenging malignancies, marked by rapid progression, early metastasis, and profound resistance to conventional approaches. Although chemotherapy continues to be the backbone of treatment, its benefit is transient and often counterbalanced by cumulative toxicity. Against this backdrop, antibody–drug conjugates have redefined therapeutic expectations. SG, the first Trop-2–targeting ADC approved for TNBC, has emerged as a prototype of precision-directed cytotoxic delivery, combining targeted efficacy with an increasingly recognized immunologic dimension.

In the context of current therapeutic standards, it is essential to distinguish sacituzumab govitecan from other first-line immunotherapy-based strategies. For patients with previously untreated PD-L1–positive (CPS ≥10) metastatic TNBC, pembrolizumab combined with chemotherapy remains the established standard regimen based on KEYNOTE-355, significantly improving survival over chemotherapy alone. In contrast, the combination of sacituzumab govitecan with pembrolizumab, despite demonstrating promising activity in trials such as ASCENT-04/KEYNOTE-D19, is still under investigation and is not currently considered a first-line standard. SG therefore occupies a later-line setting or remains an emerging option in combinations aimed at enhancing immunological synergy.

Additionally, the therapeutic landscape of breast cancer is being reshaped by the introduction of trastuzumab deruxtecan (T-DXd), which has shown substantial benefit in HER2-low disease and has expanded the use of ADCs beyond HER2-positive tumors. Although HER2-low breast cancer represents a biologically distinct group from TNBC, the success of T-DXd highlights the growing role of ADCs as a class and further contextualizes the position of SG within an evolving treatment paradigm. Together, these developments illustrate how SG integrates into a broader framework of targeted therapies and emphasize the ongoing transition from conventional chemotherapy toward biologically driven treatment strategies.

**Table 7** summarizes and compares the efficacy and safety of key therapeutic modalities used in TNBC, including chemotherapy, olaparib, SG, and immunotherapy. Notably, combination strategies integrating these approaches have demonstrated additive or even synergistic benefits, particularly when ADCs or PARP inhibitors are paired with immune checkpoint blockade, highlighting the potential for improved and more durable clinical outcomes (59). In summary, SG and immunotherapy plus chemotherapy provide superior efficacy over standard chemotherapy in appropriate TNBC populations, with distinct toxicity profiles and immunomodulatory potential unique to the immunotherapy-based regimens. These findings are supported by randomized trials and the American Society of Clinical Oncology (ASCO) guideline, underscoring the evolving role of immune-integrated and targeted strategies in TNBC management (60–62).

**Table 7:** Comparision Sacituzumab govitecan with other therapies.

Therapy	Target / mechanism	ORR	Median PFS	Key toxicities	Immunomodulatory potential	Citation
Sacituzumab govitecan (SG)	Antibody–drug conjugate (ADC) targeting Trop-2, linked to SN-38 (topoisomerase I inhibitor); bystander killing effect	30 – 35%	5.6 months	Neutropenia, diarrhea, anemia, fatigue, alopecia	Strong ICD induction, increase MHC-I, macrophage repolarization (M2 to M1), enhanced CD8+ T-cell infiltration; clear synergy with immune checkpoint inhibitors	(54)
Immunotherapy (PD-1/PD-L1 inhibitor + chemotherapy)	PD-1/PD-L1 blockade releasing effector T-cell activity; usually combined with cytotoxic chemotherapy	Around 52%	Around 9.7 months	Immune-related AEs: pneumonitis, thyroiditis, hepatitis and chemo-related toxicities - neutropenia, neuropathy	Direct immune activation; efficacy depends on PD-L1 expression, TILs, and TMB; synergistic with ICD-inducing agents	(59)
Olaparib (PARP inhibitor)	Inhibits PARP – synthetic lethality in gBRCA1/2-mutated tumors	17.1%	4.3 months	Anemia, neutropenia, nausea, fatigue; rare MDS/AML	Indirect immunomodulation via DNA damage–induced cGAS–STING activation, enhanced neoantigen load; synergistic with ICIs is under investigation	(60)
Standard chemotherapy (TPC: eribulin, vinorelbine, capecitabine, gemcitabine)	Various cytotoxic mechanisms (microtubule disruption, DNA damage, antimetabolites)	51.4%	5 months	Myelosuppression, neuropathy, nausea, vomiting, diarrhea	Weak direct immune effects; may reduce Tregs/MDSCs and release tumor antigens, enabling synergy with ICIs	(61)

This systematic review integrates evidence from five randomized and four single-arm trials, collectively establishing a consistent efficacy and safety signal for SG. The pivotal ASCENT study demonstrated a doubling of both PFS and OS versus physician’s-choice chemotherapy, with ORR exceeding 35%—a transformative achievement in a population historically refractory to systemic therapy. Comparable outcomes from subsequent analyses and meta-reviews confirm SG as the benchmark single-agent standard for pretreated TNBC. Equally important, its toxicity profile—dominated by neutropenia, diarrhea, and fatigue—remains clinically manageable and predictable, enabling sustained treatment exposure and quality-of-life preservation (59, 63).

Data from non-randomized cohorts reinforce these findings across diverse clinical contexts, including heavily pretreated and chemoresistant cases, underscoring the reproducibility of SG’s benefit beyond controlled trial environments. In particular, a recent real-world study including 381 patients reported a median age of 61 years, with 17% of patients having ECOG performance status ≥2 and 78% treated in community settings. Patients had received a median of 2 prior lines of therapy for metastatic disease. At a median follow-up of 8.7 months, median real-world overall survival (rwOS) was 11.3 months and median time to next treatment or death (TTNTD) was 5.6 months. Grade 2 and 3/4 neutropenia occurred in 25% and 27% of patients, respectively, with 59% receiving any G-CSF support. These findings indicate that SG’s efficacy and safety in routine clinical practice are consistent with results from clinical trials, while highlighting the importance of proactive management of hematologic adverse events in broader, real-world populations (64). Similarly RWE of 285 patients with unresectable locally advanced or mTNBC treated with SG across 52 oncology centers reported median PFS of 5.4 months and median OS of 12.2 months (12-month OS 49.2%), with ORR 36.8% and DCR 63.9%. Grade 3–4 adverse events occurred in 44.2% (mainly neutropenia), dose reductions were needed in 20% of cases, and no treatment-related deaths were reported (65). Although these data are encouraging, further studies are needed to confirm SG’s effectiveness across broader, more heterogeneous populations, and to explore optimal management strategies, combination regimens, and biomarker-driven patient selection (59).

Beyond its direct cytotoxicity, SG displays immune-modulating properties that expand its therapeutic relevance. By inducing immunogenic cell death and reshaping the tumor microenvironment, SG may potentiate antitumor immunity and synergize with checkpoint blockade (66). This biological rationale is increasingly validated by emerging clinical evidence. Trials such as ASCENT-04/KEYNOTE-D19 and SACI-IO TNBC demonstrated that combining SG with pembrolizumab enhances depth and durability of response, suggesting a convergence between ADC cytotoxic precision and immune reactivation. Beyond the completed trials, multiple studies are currently exploring the integration of SG with immune checkpoint inhibitors across various treatment settings. Ongoing phase II and III investigations—such as NCT04468061 (SG + pembrolizumab), NCT07040644 (SG + toripalimab), and NCT04434040 (SG + atezolizumab)—aim to determine whether combining ADC therapy with PD-1/PD-L1 blockade can further enhance tumor immunogenicity and achieve more durable responses. The results of these trials are expected to clarify the role of SG within immuno-oncology frameworks and define its optimal positioning in both metastatic and early-stage TNBC (67).

Equally compelling are data from early-stage settings. The NeoSTAR trial reported a 30% pCR rate with single-agent SG in the neoadjuvant context—an unprecedented signal for monotherapy in TNBC. Ongoing studies (NCT04230109, NCT06081244) are expected to define whether earlier integration of SG can translate into durable disease control and potential cure. Such evidence may reshape its therapeutic positioning from salvage to frontline or perioperative use.

Nevertheless, key challenges remain. Predictive biomarker discovery is imperative, as Trop-2 expression alone insufficiently predicts therapeutic benefit. Integrating genomic, immune, and transcriptomic markers—such as HRD status, TIL density, and immune gene signatures—will enable a precision-driven framework for patient selection. In parallel, the biological underpinnings of acquired resistance, including antigen loss, efflux-mediated payload clearance, and microenvironmental remodeling, warrant deeper mechanistic study.

Next-generation ADCs, exemplified by sacituzumab tirumotekan, aim to refine Trop-2 targeting through optimized linkers, improved intracellular delivery, and novel payloads. Preliminary evidence indicates enhanced potency and potential activity in SG-refractory disease, offering a glimpse into the evolution of ADC engineering.

In summary, sacituzumab govitecan has redefined therapeutic expectations in TNBC by bridging targeted cytotoxic delivery with immune modulation. Its integration into immuno-oncology paradigms, guided by biomarker-driven selection and real-world validation, represents the next frontier of precision breast-cancer therapy. Collectively, these findings position SG as both a therapeutic and immunologic bridge—capable of inducing ICD, remodeling the tumor microenvironment, and synergizing with checkpoint blockade—thus establishing a foundation for future immune-integrated treatment strategies.

## 8 Limitations

In this review, several factors should be taken into account when interpreting the results. interpreting the results. First, although the studies included comprise RCTs and single-arm clinical trials, most were single-arm with relatively small sample sizes. Moreover, the populations enrolled in these studies were highly selected, consisting primarily of patients with good performance status and limited comorbidities. These populations may not fully represent the diversity of real-world patients with TNBC, particularly those who are elderly, frail, or heavily pretreated. As a result, the generalizability of these findings to broader, real-world populations remains uncertain. RWE studies are needed to confirm the reproducibility of SG’s efficacy, safety, and tolerability in routine clinical practice.

Second, the follow-up times in many trials were short, often limited to a few months, which makes it difficult to draw conclusions about the long-term efficacy and safety of SG. In some cases, key clinical endpoints such as quality of life or duration of response were not consistently reported or compared.

Furthermore, the review only included articles published in English, which may introduce a selection bias, although no significant studies in other languages were identified during the search process. Despite these limitations, we aimed to present the current state of evidence as objectively as possible and highlight both the potential and the open questions surrounding this therapeutic approach.

## 9 Conclusions

Triple-negative breast cancer (TNBC) continues to pose a major clinical and therapeutic challenge due to its aggressive biology, high metastatic potential, and lack of molecularly targeted therapies. Sacituzumab govitecan (SG), as a Trop-2–directed antibody–drug conjugate, represents a transformative advance in precision oncology, combining potent cytotoxicity with immunomodulatory effects that reprogram the tumor microenvironment. Clinical data consistently demonstrate significant improvements in overall and progression-free survival, establishing SG as a robust alternative to conventional therapies. Beyond its monotherapy activity, SG’s dual mechanism offers a compelling rationale for combination with immune checkpoint inhibitors, potentially unlocking synergistic effects that could redefine treatment paradigms across TNBC subtypes and disease stages. Nevertheless, substantial work remains: further mechanistic studies, large-scale randomized trials, and real-world investigations are essential to refine patient selection, optimize sequencing strategies, and expand our understanding of SG’s long-term safety and efficacy across diverse populations. As ADC engineering, immuno-oncology integration, and translational research converge, SG exemplifies a model for next-generation targeted therapeutics, bridging precision medicine and immune modulation. Collectively, these advances position Trop-2–directed strategies to become a cornerstone of personalized, immune-integrated TNBC therapy, while underscoring the ongoing need for comprehensive research to fully realize their clinical potential.

## Data Availability

The original contributions presented in the study are included in the article/supplementary material. Further inquiries can be directed to the corresponding author/s.
